# Measuring retention in care for HIV-positive pregnant women in Prevention of Mother-to-Child Transmission of HIV (PMTCT) option B+ programs: the Mozambique experience

**DOI:** 10.1186/s12889-020-8406-5

**Published:** 2020-03-12

**Authors:** Laurence Ahoua, Shino Arikawa, Thierry Tiendrebeogo, Maria Lahuerta, Dario Aly, Renaud Becquet, Francois Dabis

**Affiliations:** 1 University of Bordeaux, INSERM, Bordeaux Population Health Research Center, Team IDLIC, UMR 1219, Université de Bordeaux, Case 11, 146 rue Léo Saignat, 33076 Bordeaux Cedex, France; 2 Mailman School of Public Health, ICAP at Columbia University, Rua Francisco Matange, 224/246, Caixa Postal n.°1747, Maputo, Mozambique; 3 ICAP at Columbia University, Mailman School of Public Health, Columbia University, 722 West 168th Street, New York, NY 10032 USA; 4 Department of Epidemiology, Mailman School of Public Health, Columbia University, 722 West 168th Street, New York, NY 10032 USA

**Keywords:** PMTCT, Option B +, Retention, Sensitivity, Specificity

## Abstract

**Background:**

Failure to retain HIV-positive pregnant women on antiretroviral therapy (ART) leads to increased mortality for the mother and her child. This study evaluated different retention measures for women’s engagement along the continuum of care for prevention of mother-to-child transmission (PMTCT) option B+ services in Mozambique.

**Methods:**

We compared ‘point’ retention (patient’s presence in care 12-month post-ART initiation or any time thereafter) with the following definitions: alive and in care 12 month post-ART initiation (Ministry of Health; MOH); attendance at a health facility up to 15-month post-ART initiation (World Health Organization; WHO); alive and in treatment at 1-, 2-, 3-, 6-, 9-, and 12-month post-ART initiation (Inter-Agency Task Team; IATT); and alive and in care 12-month post-ART initiation with ≥75% appointment adherence during follow-up (i.e. ‘appointment adherence’ retention) or with ≥75% of appointments met on time during follow-up (i.e. ‘on-time adherence’ retention). Kaplan-Meier survival curves were produced to assess variability in retention rates. We used ‘on-time adherence’ retention as our reference to estimate sensitivity, specificity, and proportion of misclassified patients.

**Results:**

Considering the ‘point’ retention definition, 16,840 HIV-positive pregnant women enrolled in option B+ PMTCT services were identified as ‘retained in care’ 12-month post-ART initiation. Of these, 60.3% (95% CI 59.6–61.1), 84.8% (95% CI 84.2–85.3), and 16.4% (95% CI 15.8–17.0) were classified as ‘retained in care’ using MOH, WHO, and IATT definitions, respectively, and 1.2% (95% CI 1.0–1.4) were classified as ‘retained in care’ using the ‘≥75% on-time adherence’ definition. All definitions provided specificity rates of ≥98%. The sensitivity rates were 3.0% with 78% of patients misclassified according to the WHO definition and 4.3% with 54% of patients misclassified according to the MOH definition. The ‘point’ retention definition misclassified 97.6% of patients. Using IATT and ‘appointment adherence’ retention definitions, sensitivity rates (9.0 and 11.7%, respectively) were also low; however, the proportion of misclassified patients was smaller (15.9 and 18.3%, respectively).

**Conclusion:**

More stringent definitions indicated lower retention rates for PMTCT programs. Policy makers and program managers should include attendance at follow-up visits when measuring retention in care to better guide planning, scale-up, and monitoring of interventions.

## Background

In 2015, the World Health Organization (WHO) recommended that all HIV-positive pregnant women be provided with lifelong antiretroviral therapy (ART) regardless of their CD4 count and WHO staging, also called “option B+” [1]. Compared to previous prevention of mother-to-child transmission (PMTCT) of HIV regimens, such as option A (short term anti-retroviral prophylaxis) or option B (triple anti-retroviral until end of breastfeeding) [2], option B+, by providing the same antiretroviral triple-combination to all HIV-positive pregnant women for life, has the advantages of lowering the risk of HIV transmission to the male partners since being on ART will make women less infectious, reducing the risk of MTCT of HIV in subsequent pregnancies while reducing the risk of child infection to less than 5% in resources limited settings [1].

Retention in PMTCT care has both individual and public health implications. For mother-infant pairs, being in care contributes to higher maternal ART adherence, better viral suppression, and ensures better post-natal care, including full ART prophylaxis and complete infant testing for HIV-exposed infants (HEI) [3, 4]. In contrast, failure to be retained in care can lead to delayed or inconsistent use of antiretroviral medications, higher risk of maternal viral load failure, and increased morbidity and mortality for the mother and her child [5–7]. In addition, recent publications have reported an upward trend in acquired drug resistance to first-line ART across low- and middle-income countries, principally due to sub-optimal viral suppression [8–10]. Therefore, non-achievement of viral suppression can lead to a higher risk of HIV transmission and secondary infections with acquired drug resistance strains, and is therefore considered a public health threat [11].

When referring to retention in care, publications often consider ‘point’ retention, which is defined in relation to a patient’s presence in care at a certain time point [12]. For women and children enrolled in PMTCT services, attendance at a clinic at a certain time point is often considered to be full retention over this time period. While such a simple definition is useful, it has been demonstrated that between one- and two-thirds of HIV-infected adult patients are not in regular care [13]. Therefore, regularity of attendance is an important parameter to consider if the quality of engagement in care is to be evaluated [14].

To address the shortcomings in this simple definition, attempts have been made to better measure retention in care in PMTCT services [15, 16]. In 2014, a WHO monitoring and evaluation working group published consolidated strategic information guidelines and proposed that retention in care of HIV-infected pregnant and breastfeeding women was equivalent to attendance at a health facility at 12-month post-initiation of ART, or at any time up to 3 months later [15]. The 12-month time point was agreed to align with the adult ART monitoring guidelines; however, it fails to address HIV transmission risk beyond 12 months due to prolonged breastfeeding. In 2015, the Inter-Agency Task Team (IATT**)** on Children and HIV and AIDS defined maternal retention as ‘the proportion of HIV-positive pregnant and/or breastfeeding women on ART alive and in treatment at 1, 2, and 3 months post-ART initiation (early retention), and then at 6, 9, and 12 months post-ART initiation’, where retention was considered as a continuous engagement [16]. Despite such efforts, there is no consensus on what constitutes ‘retention in care’, and no gold standard has been determined to date. In 2010, Mugavero et al. provided a synopsis of five commonly used definitions of retention in HIV care and treatment services, ranging from a simple count of the number of missed visits to a more complex medical visit performance measure that incorporated elements of appointment consistency and gaps in care [13]. While that study provided important insights on methodological and conceptual strengths and limitations of each definition, no comparative analysis of these definitions has been undertaken. We hypothesized that ‘point’ retention might overestimate the level of engagement in care of women enrolled in PMTCT programs, giving a false image of clinical and programmatic success. In a high HIV-burden country such as Mozambique, women’s engagement should be more precisely assessed in order to identify windows of opportunity for possible improvement throughout the PMTCT care continuum and to better account for the number of children in need of ART [17].

In this study, we aimed to answer the following questions: What is the effect of different definitions of retention in care on the interpretation of women’s engagement along the PMTCT continuum of care programs under option B+ in Mozambique? What are the advantages and limitations of each definition? Finally, what other measures of retention besides ‘point’ retention could more accurately reflect the nature of women’s engagement along the PMTCT care continuum? Specifically, this study i) assessed the variability of different measures of retention at 12 months post-ART initiation under PMTCT option B+ programs using six different definitions of retention in care, (ii) compared the sensitivity and specificity of the different definitions in detecting women fully engaged in care at 12 months post-ART initiation, and (iii) discussed the programmatic implications of each definition in the context of PMTCT option B+ programs.

## Methods

### Study design

This was a retrospective cohort study involving secondary analysis of routinely collected data.

### Setting

Data were extracted from all sites with available electronic patient-level databases (ePLD), representing a total of 86 Ministry of Health (MOH) facilities in Nampula and Zambézia Provinces in Mozambique that were included in the analysis. All of these sites provided PMTCT option B+ and HIV care and treatment services with the support of ICAP at Columbia University through funding from the United States President’s Emergency Plan for AIDS Relief (PEPFAR).

Officially adopted in 2013, the option B+ strategy in Mozambique uses a ‘one-stop-model’ and ‘Test and Treat’ approach, wherein pregnant and breastfeeding women are counseled and tested in mother and child health (MCH) services and, if found HIV-positive, are started on lifelong ART that same day. HIV care and treatment are integrated within MCH services and ART provision is administered by nurses. For HIV-positive pregnant women, a second consultation occurs within the first week post-ART initiation, and a monthly clinical follow-up is conducted during the first 6 months of ART, followed by bi-monthly follow-ups during breastfeeding until 12 months of treatment and bi-annually thereafter until the end of breastfeeding. ART counseling and drug pickup are conducted on a monthly basis for the first year of treatment. Routine viral load (VL) monitoring is recommended at 3 and 12 months post-ART initiation and annually thereafter. HIV-positive women are followed-up in MCH services until the final HIV status of the exposed infant is determined [18]. More detailed descriptions of the PMTCT program settings and data sources are described elsewhere [19].

The Mozambique provinces targeted for the study officially implemented option B+ in July 2013. Of all HIV-positive women who were enrolled in PMTCT option B+ programs and who were started on ART between July 1, 2013 and December 31, 2017, we selected those who were considered as ‘retained in care’ at 12 months post-ART initiation according to the ‘point’ retention definition (i.e. patient alive and present in care at 12 months post-ART initiation, or any time thereafter) and with a follow-up time of at least 12 months under ART to allow complete assessment of engagement in care during the first year of treatment. These selected women constituted the basis of comparison for the subsequent analyses assessing variability in different definitions concerning retention measures.

### Comparing definitions

The ‘point’ retention definition was compared with the following five definitions: MOH [20], WHO [15], IATT [16], ‘appointment adherence’ retention [13], and ‘on-time adherence’ retention [21] (Table 1). Similar to the ‘point’ retention definitions, the MOH and WHO retention definitions assess retention at a single time point. For women’s attendance at a clinic, we considered different types of visits, including medical consultations, pharmacy drug refills, counseling, or laboratory analysis. This approach allowed us to consider HIV care as a holistic strategy, given that HIV care is provided in an increasingly team-based environment with non-prescribing healthcare professionals taking on expanded roles in direct patient care. Table 1 summarizes the definitions of retention according to different measures and shows how failure events were accounted for in statistical analyses.

**Table 1:** Retention definitions and methods of calculation. B+ PMTCT program, Mozambique, 2013–2017

Type of retention	Definition of a patient ‘alive and retained in care’ at 12 months post-ART initiation	Failure event
‘Point’ retention	Alive and had a visit at the health facility 12 months post-ART initiation or if was known to have had a visit at a health facility any time after	Death, LTFU, and transfer-out are counted if they occurred within the first 12 months post-ART initiation
WHO	Attendance at a health facility at 12 months post-ART initiation^a^, or at any time up to 3 months later	Death, LTFU, and transfer-out are counted if they occurred within the first 15 months post-ART initiation. Patients transferred out were right-censored at the date of transfer-out
MOH	Attendance at a health facility at 12 months post-ART initiation^a^	Death or LTFU are counted if either occurred within the first 12 months post-ART initiation Patients transferred out are excluded from the analysis
IATT	Attendance at a health facility and on treatment at 1, 2, 3, 6, 9, and 12 months post-ART initiation ^b^	Death, LTFU, transfers-out and failure to attend either the 1-, 2-, 3-, 6-, 9-, or 12-month visit, whichever comes first Patients are right-censored at the date of the 1st failure event.
‘Appointment adherence’ retention	Attendance at a health facility at 12-months post-ART initiation ^a^*and* ≥ 75% of appointment adherence to scheduled visits ‘Appointment adherence’ was estimated using the number of visits attended divided by the number of total scheduled visits during the 12-month observation period	Not applicable
‘On-time adherence’ retention	Attendance at a health facility at 12 months post-ART initiation ^a^*and* ≥ 75% of ‘on-time attendance to scheduled visits A visit on time is defined as a visit that occurred within +/− 15 days of the date of the expected scheduled visit ‘On-time adherence’ is estimated using the number of visits attended on-time divided by the number of total scheduled visits. Unscheduled visits occurring before the date of appointment were not counted as missed visits	Death, LTFU, transfer-out, and failure to attend a visit on-time, whichever comes first. Patients were right-censored at the date of the 1st failure event

*ART* antiretroviral therapy, *IATT* Inter-Agency Task Team, *LTFU* lost to follow-up, *MOH* Ministry of Health, *PMTCT* for prevention of mother-to-child transmission, *WHO* World Health Organization

^a^For these definitions, we considered a window period of +/− 15 days around the theoretical date of 12-month post-ART initiation

^b^ We allowed a +/− 15-day window period for each stage of ART follow-up. Note: We did not consider ‘appointment adherence’ retention for the survival analysis, as this definition does not contemplate a time event but rather the total number of visits completed during the observation period

### Statistical analysis

A total of 31,186 HIV-positive women enrolled in the PMTCT option B+ program in 86 MOH facilities in Nampula and Zambézia Provinces and initiated ART during the study period. Women’s appointment adherence, as well as their on-time adherence to scheduled visits, were expressed as percentages, median, and interquartile range [IQR]. Women’s retention status was classified into a binary outcome (retained vs. not retained in care) using a 75% threshold for clinical attendance. Each woman contributed to the analyses from the date of ART initiation to the first occurrence of a failure event, as described in Table 1. To allow sufficient time to analyze clinical attendance within the first year of treatment, we only included women who initiated ART up until December 31, 2016 and for whom the theoretical follow-up time under ART was ≥12 months.

Kaplan-Meier survival curves were produced to estimate retention in care at 3, 6, 9, and 12 months post-ART initiation using each of the definitions described above. We assessed variability in the different measures of retention between respective definitions used. We did not consider ‘appointment adherence’ retention for the survival analysis, as this definition does not contemplate a time event, but rather a total number of visits completed during the observation period.

We assessed sensitivity, specificity, and the proportion of misclassified women for each alternative definition of retention compared to the reference of an ‘ideal case scenario’ of full ‘on-time adherence’ retention, defined as a woman alive and in care 12 months post-ART initiation and ≥ 75% of scheduled visits attended on time (+/− 15 days). Sensitivity and specificity values were calculated with 95% confidence intervals.

## Results

Of 31,186 HIV-positive women enrolled in the PMTCT option B+ program during the study period, 18,739 were considered as being retained in care at 12 months post-ART initiation according to the ‘point’ retention definition. We excluded 1899 women with a follow-up ART time of < 12 months. Finally, 16,840 women were included in this study.

**Fig. 1:**
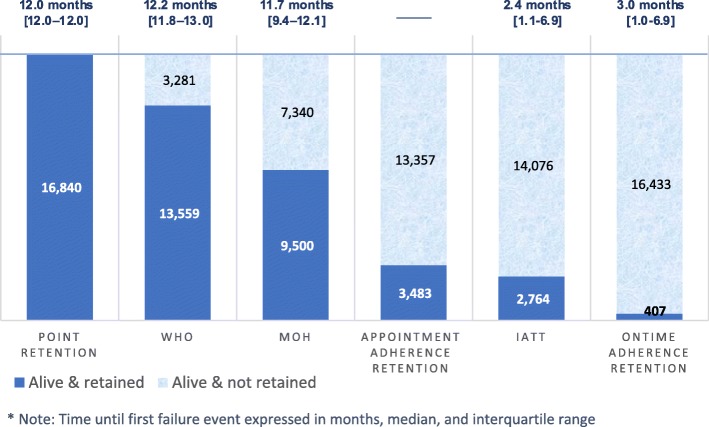
B+ pregnant women retained in care 12 months post-ART initiation: time to first failure event

Of 16,840 women included, only 2764 (16%) and 407 (2%) were considered retained at 12 months post-ART initiation when considering regularity and timeliness of appointment attendance, respectively (Fig. 1). The occurrence of irregular (IATT definition) and delayed (‘on-time’ definition) clinical attendance was documented as early as 2–3 months post-ART initiation. For all women included, the median appointment adherence was 66.7% (IQR 57.1–80.0%) and the median on-time adherence was 40.0% (IQR 22.2–52.9%) during the 12-month observation period.

**Fig. 2:**
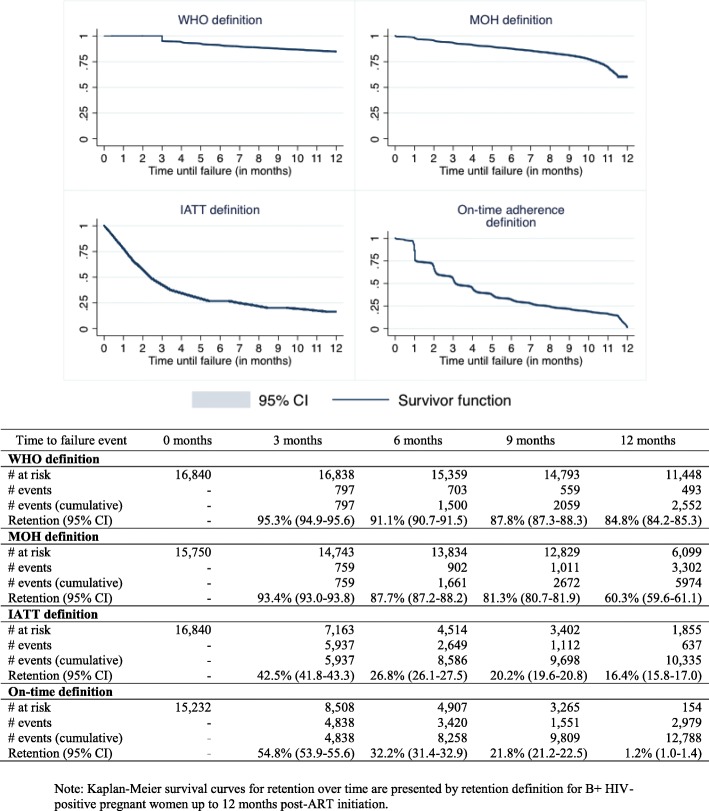
Estimated Kaplan-Meier survival curves for retention over time, by retention definition

Figure 2 shows Kaplan-Meier survival curves for retention over time up to 12-month post-ART initiation using the different definitions. Among the 16,840 B+ pregnant women considered retained in care according to the ‘point’ retention definition, 84.8% (95% CI 84.2–85.3) were actually defined as retained in care when estimated using the WHO definition (i.e. attendance at a health facility up to 15 months post-ART initiation), 60.3% (95% CI 59.6–61.1) with the MOH definition (i.e. alive and in care at 12 months post-ART initiation), and 16.4% (95% CI 15.8–17.0) with the IATT definition (i.e. alive and on treatment at 1, 2, 3, 6, 9, and 12 months post-ART initiation), respectively. When using the ‘on-time adherence’ retention definition (i.e. alive and in care at 12 months post-ART initiation with at least 75% of on-time adherence during follow-up), only 1.2% (95% CI 1.0–1.4) were defined as retained in care.

We considered the reference category to be the 407 women alive and in care 12-months post-ART initiation with at least 75% on-time adherence. We calculated the sensitivity and specificity of other definitions to more accurately classify the women into a more suitable category of retention and determine the proportion of misclassified patients. While all definitions provided specificity rates of ≥98%, the sensitivity to detect a patient actually retained in care with ≥75% of on-time adherence was < 12% (Table 2). The WHO and MOH retention definitions provided the lowest sensitivity figures and the highest proportion of women misclassified as retained in care, as follows: 3.0% sensitivity (95% CI 2.7–3.3%) with 78% of patients misclassified according to the WHO definition and 4.3% sensitivity (95% CI 3.9–4.7%) with 54% of patients misclassified according to the MOH definition. In addition, the ‘point’ retention definition misclassified 97.6% of patients. Although sensitivity rates were also low when using the IATT and ‘appointment adherence’ retention definitions, the proportion of misclassified patients was relatively smaller (15.9 and 18.3%, respectively).

**Table 2:** Sensitivity and specificity of various definitions of retention in care among B+ HIV+ pregnant women defined as alive and retained in care according to the ‘point’ retention definition (*n* = 16,840)

Retained in care according to type of definition (*n* = 16,840)	Alive and retained in care with ≥75% of visits in time (Reference)		Sensitivity 95% CI	Specificity 95% CI	% of misclassification
	Yes	No			
‘Point’ retention	407	16,433	–	–	97.6%
WHO	405	13,154	3.0% [2.7–3.3%]	99.9% [99.8–100%]	78.1%
MOH	407	9093	4.3% [3.9–4.7%]	100% [99.9–100%]	54.0%
IATT	249	2515	9.0% [8.0–10.1%]	98.9% [98.7–99.0%]	15.9%
‘Appointment adherence’ retention	407	3076	11.7% [10.6–12.8%]	100% [100–100%]	18.3%

The ‘on-time adherence’ definition of retention is considered as the reference (*n* = 407 women retained in care at 12-months post-ART initiation and with ≥75% on-time adherence to scheduled visits). The percentage of misclassified patients is calculated using the sum of patients incorrectly classified according to each definition divided by the total number of patients included in the analysis

*CI* confidence interval, *IATT* Inter-Agency Task Team, *MOH* Ministry of Health, *WHO* World Health Organization

## Discussion

We analyzed six different definitions of ‘retention in care’ for HIV-positive pregnant women enrolled in PMTCT option B+ programs, ranging from the most lenient definition using a ‘point’ retention approach to the most stringent definition using an ‘on-time adherence’ retention approach. Of the six definitions, three assess retention at a single time point (‘point’ retention, WHO, and MOH). The IATT, ‘appointment’, and ‘on-time adherence’ retention definitions reflect a more continuous engagement in care, in which follow-up visits between initiation of ART and the endpoint date are considered.

As expected, we found that the more stringent the definition, the lower the probability for women to be defined as retained in care; when levels of attendance at visits during follow-up were considered, estimates were even lower (85 and 60% when using the WHO and MOH definitions compared to 16 and 1% when using the IATT and ‘on-time adherence’ retention definitions). At the end of the first year, most women starting ART had not adhered to their scheduled visits, with median appointment adherence at 66% and median on-time adherence at 40% among the 16,840 women considered as retained in care using the ‘point’ retention definition. Of these, only 16 and 2% were actually defined as retained in care 12 months post-ART initiation if a threshold of ≥75% for appointment and on-time attendance were considered, respectively.

Initial visits in the first year of ART initiation are critical to ensure successful engagement in care, as they convey important preventive healthcare and risk reduction messages, involve intensive counseling, peer support, and monitor patterns of healthier behavior for the women and their exposed children. This interaction between the health system and the HIV-infected woman determines the survival likelihood of the pair and the mother-to-child transmission of HIV. Adding the frequency and regularity of visits into the concept of retention increases the quality of the PMTCT program evaluations, enabling a distinction to be made between women who attend for all, or some care and treatment, and those who completely fail to attend scheduled visits and are therefore considered not to be engaged in care.

Table 3 provides a summary of the advantages, limitations, and programmatic implications of the six measures of retention for PMTCT option B+ programs. Based on our results, the three-single time point measures, namely, the ‘point’, WHO, and MOH definitions, were classic programmatic approaches for measuring retention in care under ART. While relatively easy to use, they do not capture important milestones in the PMTCT context; for example, at delivery, at 2-month post-partum for early infant diagnosis, or at the end of breastfeeding for the final HIV determination of the HEI. In addition, they do not consider visit consistency, which has been demonstrated to be significantly associated with ART adherence and viral suppression among HIV-positive adults [22, 23]. However, the three definitions that capture visit consistency along the continuum of care, namely, the IATT, ‘appointment’, and ‘on-time adherence’ definitions, were more complex to use as they included multiple clinic visits (repeated measures) occurring longitudinally over time. This could become extremely challenging in limited-resource settings where limited qualified human resources are available to document such information, data collection tools are not adapted to capture longitudinal follow-up, and there are numerous entry points where women are followed-up within the same health facility or between sites [14, 24]. To correctly capture patients’ follow-up, different data sources need to be combined or triangulated, for example, with counseling registers and laboratory or pharmacy records. Electronic databases may help in this process, but these are generally implemented in high volume, accessible sites and may not necessarily be representative of the entire country for national programs. However, all three definitions enabled adding the concept of a continuum of care to point retention estimates.

**Table 3:** Advantages, limitations, and programmatic practicality of six measures of retention in care for PMTCT option B+ programs

Retention definition	Advantages	Limitations	Programmatic practicality
‘Point’ retention	Easy to measure Assessed at a single time point Provide a transversal picture of retention	Does not consider visit consistency of the MIP Does not consider whether the women fully adhered to the 12-month visit schedule Not aligned with important PMTCT milestones (i.e. delivery, EID, or final HIV testing)	Programmatic definition of retention Achievable with simple health information systems (e.g. paper-based registers)
WHO	Easy to measure Assessed at a single time point Provide a transversal picture of retention More specific in detecting patients adhering to the 12-month visit (up to 15-months only)	Does not consider visit consistency of the MIP Not aligned with important PMTCT milestones (i.e. delivery, EID, or the end of breastfeeding)	As above
MOH	Easy to measure Assessed at a single time point Provides a transversal picture of retention Considers whether the women fully adhered to the 12-month visit schedule	As above	Programmatic definition of retention Achievable with simple health information systems (e.g. paper-based registers) Cohort based approach for calculation
IATT	Considers whether the women fully adhered to the 12-month visit schedule Captures visit consistency Can be adapted to align with the follow-up of the MIP	More complex to measure (ideally requires an ePLD or POC EMR) Not systematically aligned with national PMTCT follow-up guidelines but can be modified accordingly Probable need of data triangulation with other data sources (e.g. pharmacy, laboratory) and linkage with unique IDN	Relevant in capturing visit consistency of the MIP, can be aligned with important PMTCT milestones Ideal if integrated POC testing services for the MIP are available Better alternative than single time point estimations
‘Appointment adherence’ retention	Considers whether the women truly adhered to the 12-month visit schedule Capture visit consistency Measurable with paper-based longitudinal cohort based registers (total # of completed visits done/total scheduled visits)	Does not capture the regularity or timeliness of completed visits Not aligned with important PMTCT milestones for the MIP Highly dependent on data completeness of denominator (# of scheduled visits) Need of data triangulation with other data sources (e.g. pharmacy, laboratory) and linkage with unique IDN	Achievable with simple health information systems Better alternative than single time point estimations
‘On-time adherence’ retention	Considers whether the women fully adhered to the 12-month visit schedule Ideal to capture correct levels of engagement in care for PMTCT (regularity and timeliness) Aligned with important milestones of PMTCT follow-up for the MIP	More complex to measure (requires an ePLD or POC EMR) Highly dependent on data completeness of denominator (# of scheduled visits) Need of data triangulation with other data sources (e.g. pharmacy, laboratory) and linkage with unique IDN Time consuming activity not compatible with one-stop model PMTCT services in the absence of electronic databases	Adapted for research purposes Not compatible with routine monitoring of retention in care of MIPs

*EID* early infant diagnosis, *EMR* electronic medical record, *ePLD* electronic patient-level database, *IATT* Inter-Agency Task Team, *IDN* identification number, *MIP* mother-infant pair, *MOH* Ministry of Health, *POC* point-of-care, *PMTCT* for prevention of mother-to-child transmission, *WHO* World Health Organization

When considering ‘on-time adherence’ retention as the reference, all other definitions provided very low sensitivity rates to accurately detect patients retained in care, with high rates of misclassified patients. The WHO and MOH retention definitions provided the lowest sensitivity rates, of 3 and 4%, respectively. For the ‘point’ retention, WHO, and MOH definitions, which do not consider continuous follow-up in their calculations, the proportion of misclassified patients ranged from 54 to 97%. The ‘appointment adherence’ definition provided the highest sensitivity rate (11.7%), with a fairly low proportion of misclassified patients (18.3%) compared to all other definitions analyzed. We found that the median time to first failure of correct follow-up was short (≤3 months), if visit attendance during follow-up was considered, which demonstrates the need to implement early measures to prevent patients in the PMTCT program from disengaging from care. Such strategies should focus on already well-known barriers to regular attendance, such as a lack of disclosure, poor staff attitudes, competing personal priorities, medication side-effects, or transportation difficulties [21].

While our definition of ‘on-time adherence’ retention represents the ideal situation, in which an HIV-positive pregnant woman who enrolls in a PMTCT program and starts ART under option B+ should be considered as fully engaged in care to ensure an optimal viral response, it is perhaps too stringent for PMTCT program evaluations. This definition is also time consuming to use on a routine basis and may not be compatible with current PMTCT service settings. Therefore, we recommend using either the IATT or the ‘appointment adherence’ retention definitions to better measure levels of engagement in care for mother-infant pairs.

Our analysis had several limitations. To accurately evaluate attendance at scheduled visits, it is desirable to distinguish visits canceled in advance (either by the patient or the care provider) from ‘no show’ visits that are missed by the patient [13]. Our data did not allow for this distinction to be made, as this information was not captured in the database. Therefore, our results may have underestimated the real values of women’s engagement in care when analyzing ‘appointment’ and ‘on-time’ adherence retentions.

Some women, although categorized as having full attendance at scheduled visits, did not follow per se the recommended schedule of visits according to national guidelines. More frequent visits may indicate a more advanced HIV disease, problems in counseling that need to be addressed, or drug or laboratory reagent stock-outs [24]. Less frequent clinical visits may reflect a stabilized disease stage or temporary silent transfers (i.e. women receiving ART services elsewhere for a short period of time). For this reason, we chose to use all scheduled visits, regardless of whether the visits fell into expected time intervals. For similar reasons, we did not analyze treatment interruptions and gaps in care. Further research is warranted to investigate the effect of a lack of observation of the recommended schedule of visits as per national guidelines on PMTCT program outcomes.

Finally, we analyzed data from health facilities with ePLD in two provinces in Mozambique. Although these two provinces are among the most populated in the country, with large volumes of patients seen at health facilities, our results may not be generalizable to other regions in Mozambique or to other resource-limited settings.

## Conclusion

Our findings suggest that women’s engagement to care cannot be precisely assessed without taking into account their presence at key time-points along the continuum of care. Lack of agreed definition hinders comparison of findings from different programs. We recognize that capturing timeliness and regularity of women’s clinical attendance with routine data can be a complex task and that applying more stringent definitions is likely to result in lower rates of retention in care in PMTCT programs. Nonetheless, HIV services should improve its monitoring systems to ensure that clinical decision be made based on sound and timely assessment of women’s retention and key indicators related to maternal viral load, infant prophylaxis or infant HIV serostatus determination. A consensus on universal definition is urgently needed; however, data availability, diverse clinical practices in different settings, and local analytical capacities should be taken into account to ensure practicability of the definition. Since retention estimates are used for the modeling of vertical transmission rates of HIV, lack of precision could lead to false interpretation of program achievements in relation to elimination of mother-to-child transmission of HIV. This consideration may become particularly important as recent data seems to indicate a stagnation in the achievements of PMTCT programs worldwide, which suggests the need for revised global, national, and local strategies to end the HIV epidemic by 2030.

## Data Availability

No original data were used in the study; only secondary, routinely collected, de-identified, service delivery data were used. The datasets analyzed during the current study are currently not publicly available. An anonymized dataset can be available from the corresponding author on reasonable request.

## Abbreviations

ART: Antiretroviral treatment
ePLD: Electronic patient-level database
HEI: HIV-exposed infants
IATT: Inter-Agency Task Team
IQR: Interquartile range
MCH: Mother and child health
MOH: Ministry of Health
PEPFAR: United States President’s Emergency Plan for AIDS Relief
PMTCT: Prevention of mother-to-child transmission of HIV
VL: Viral load
WHO: World Health Organization
